# TachoSil^®^ for prevention of postpancreatectomy hemorrhage after distal pancreatectomy: a propensity-weighted pilot study

**DOI:** 10.1007/s00423-026-04022-5

**Published:** 2026-03-23

**Authors:** Claudio Ricci, Vincenzo D’Ambra, Federico Pisani, Laura Alberici, Carlo Ingaldi, Marco Fichera, Riccardo Casadei

**Affiliations:** 1 Department of Internal Medicine and Surgery (DIMEC); Alma Mater Studiorum, University of Bologna, Pancreas Unit, Bologna, 40138 Italy; 2 Division of Pancreatic Surgery, IRCCS Azienda Ospedaliero-Universitaria Di Bologna, via Albertoni 15, Bologna, Italia

**Keywords:** pancreatic surgery, hemorrhage, absorbable fibrin sealant patch

## Abstract

**Purpose:**

Postpancreatectomy hemorrhage (PPH) is a rare but potentially lethal complication after distal pancreatectomy (DP), often precipitated by clinically relevant postoperative pancreatic fistula (POPF). While the fibrin sealant patch TachoSil^®^ has been evaluated for POPF prevention, its role in mitigating PPH remains underexplored.

**Methods:**

We conducted a single-center, retrospective cohort study of consecutive adult patients undergoing DP (2015–2024). Patients were classified by intraoperative use of TachoSil^®^ in addition to standard stump closure. The primary endpoint was PPH, defined per International Study Group of Pancreatic Surgery (ISGPS) criteria. Secondary endpoints included clinically relevant POPF, Comprehensive Complication Index (CCI), reoperation, 90-day mortality, and length of stay (LOS). Inverse probability of treatment weighting (IPTW) was applied to adjust for baseline covariates.

**Results:**

Of 224 patients, 56 (25%) received TachoSil^®^. After IPTW adjustment, TachoSil^®^ was associated with a reduced risk of PPH (RD − 0.065; 95% CI − 0.121 to − 0.009; *p* = 0.014), corresponding to a number needed to treat (NNT) of 15 and an E-value of 6.7. No significant differences were observed for clinically relevant POPF (RD 0.116; *p* = 0.105), CCI (MD 3.459; *p* = 0.091), reoperation (RD − 0.016; *p* = 0.486), 90-day mortality (RD 0.012; *p* = 0.498), or LOS (MD − 0.242 days; *p* = 0.856). Stratified analysis showed a pronounced reduction in PPH among patients who developed POPF (RD − 0.180; 95% CI − 0.311 to − 0.048; *p* = 0.004; NNT = 6; E-value = 12.6), while no significant effect was observed in patients without POPF.

**Conclusions:**

TachoSil^®^ use after DP was not associated with lower POPF rates but significantly reduced PPH incidence, particularly in patients developing POPF. These findings support selective TachoSil^®^ application in high-risk patients as part of a targeted strategy to prevent severe hemorrhagic complications after DP.

## Introduction

Distal pancreatectomy (DP) is the standard surgical treatment for both benign and malignant body-tail pancreatic neoplasms. Despite advances in surgical technique and perioperative care, morbidity after DP remains substantial. The clinically relevant postoperative pancreatic fistula (POPF) [1] is the most frequent complication, occurring in 20%–40% of cases in high-volume series. [2, 3] However, POPF is detrimental not only to itself, but also because it precipitates other complications such as hemorrhage and sepsis. Among these, postpancreatectomy hemorrhage (PPH) is one of the most feared, with reported mortality rates up to 50% in severe cases. [4, 5] PPH is often secondary to POPF-induced vascular erosion or pseudoaneurysm formation, and its management frequently requires complex interventional radiology or reoperation, thereby prolonging hospitalization and increasing costs. [6, 7] Several strategies have been proposed to reduce the risk of PPH after pancreaticoduodenectomy (PD) [8], while the event is underestimated in DP. Some years ago, an Italian multicenter randomized controlled trial (RCT) by Montorsi et al. [7] was conducted to investigate the benefit of an absorbable fibrin sealant patch (TachoSil^®^) in addition to standard pancreatic stump closure. This study failed to demonstrate the reduction of the POPF rate.

However, at the time of the study, no standardized definition of PPH was available, precluding systematic assessment of this outcome across studies and potentially leading to underreporting. Since then, the ISGPS has provided a consensus definition for PPH, enabling consistent classification and comparison across studies. [4] This has opened the door to re-evaluating interventions not merely for their ability to reduce POPF incidence, but for their impact on preventing high-risk, clinically meaningful complications such as PPH.

In this context, this is an explorative study that aims to evaluate the effect of TachoSil^®^ following DP, with a specific emphasis on its potential role in mitigating PPH, using standardized contemporary definitions and robust adjustment for confounding. For this purpose, we applied the inverse probability of treatment weighting (IPTW) to emulate the balance of a randomized trial while preserving the granularity of real-world surgical practice.

## Methods

This was a single-center, retrospective analysis of a prospectively maintained pancreatic surgery database at the IRCCS Azienda Ospedaliero–Universitaria di Bologna, Sant’Orsola–Malpighi Hospital. All consecutive adult patients undergoing distal pancreatectomy between January 2015 and December 2024 were considered. The study protocol was approved by the local Ethics Committee (Comitato Etico di Area Vasta Emilia Centro, PANBO 064/2017/U/Oss), and informed consent for data collection and analysis was obtained from all participants.

### Data source and variables

Baseline demographic and clinical data extracted from the database included: age, sex, body mass index (BMI), American Society of Anesthesiologists (ASA) class, comorbidities (cardiovascular, pulmonary, metabolic, hematologic), preoperative symptoms, and preoperative antiplatelet or anticoagulant therapy. Operative variables comprised surgical approach (open or laparoscopic), type of resection (body-tail or neck-body-tail), splenectomy, vascular or extended resection, pancreatic stump characteristics (Wirsung size and texture), and closure technique (hand-sewn or stapler), operative time, estimated blood loss, and use of additional hemostatic agents other than TachoSil^®^. All procedures were performed by experienced pancreatic surgeons who had already completed the learning curve for distal pancreatectomy in a high-volume pancreatic surgery unit. All patients undergoing distal pancreatectomy received a single prophylactic Jackson-Pratt closed and with active suction drain placed at the pancreatic stump.

### Endpoints and definitions

The primary endpoint was postpancreatectomy hemorrhage (PPH) according to the International Study Group of Pancreatic Surgery (ISGPS) definition (2007), which classifies PPH into three grades (A–C) based on timing, clinical impact, and site of bleeding. [4] The secondary endpoints included: clinically relevant POPF (grades B/C) as defined by the updated ISGPS criteria (2016) [1] ; Comprehensive Complication Index (CCI), summarizing all postoperative complications weighted by severity [9]; reoperation rate within 90 days; 90-day mortality; postoperative length of stay (LOS, in days).

Patients were classified into two groups according to the use of TachoSil^®^ on the pancreatic remnant at the end of resection, in addition to standard closure.

### Statistical analysis

To account for baseline differences between groups, inverse probability of treatment weighting (IPTW) was applied using propensity scores derived from a multivariable logistic regression model including all clinically relevant covariates (age, sex, BMI, ASA class, comorbidities, surgical approach, type of resection, splenectomy, vascular resection, extended resection, stump closure technique, operative time, and use of other hemostatic agents). IPTW was applied using propensity scores to adjust for differences in baseline covariates between treatment groups. Intraoperative decisions regarding TachoSil^®^ use were made by the operating surgeons based on known or unknown risk factors. IPTW creates a weighted pseudo-population in which measured confounders are balanced, thereby reducing the risk of selection bias when estimating the effect of TachoSil^®^ on postpancreatectomy hemorrhage Covariate balance before and after weighting was assessed using standardized mean differences (SMD), with values ≤ 0.2 indicating acceptable balance. [10] The standardized mean difference (SMD) is a unitless measure of effect size used to quantify differences in baseline covariates between groups. Interpretation thresholds proposed initially by Cohen classify SMD values as follows: <0.2 = small, 0.2–0.5 = moderate, 0.5–0.8 = large, and ≥ 0.8 = very large. [11]

Risk differences (RD) with 95% confidence intervals (CI) were calculated for binary outcomes, and mean differences (MD) for continuous outcomes. For outcomes with statistically significant associations, the number needed to treat (NNT) was estimated as the inverse of the RD. [12] For each significant association, the e-value was also computed to assess the potential impact of unmeasured confounding. [13] The e-value represents the minimum strength of association, on the risk ratio scale, that an unmeasured confounder would need to have with both the exposure and the outcome, beyond the measured covariates, to explain away the observed effect fully. No universally accepted thresholds exist. However, in surgical and epidemiologic literature, values close to 1 indicate low robustness, values between 1.5 and 2 suggest moderate robustness, and values > 2 are generally considered robust to residual confounding, with > 3–4 indicating extreme robustness. [13].

All analyses were conducted in Stata (version 19, StataCorp LLC, College Station, TX). Statistical significance was set at *p* < 0.05 (two-tailed).

## Results

A total of 224 patients underwent distal pancreatectomy, of whom 56 (25.0%) received TachoSil^®^ and 168 (75.0%) did not.

### Baseline characteristics

**Fig. 1:**
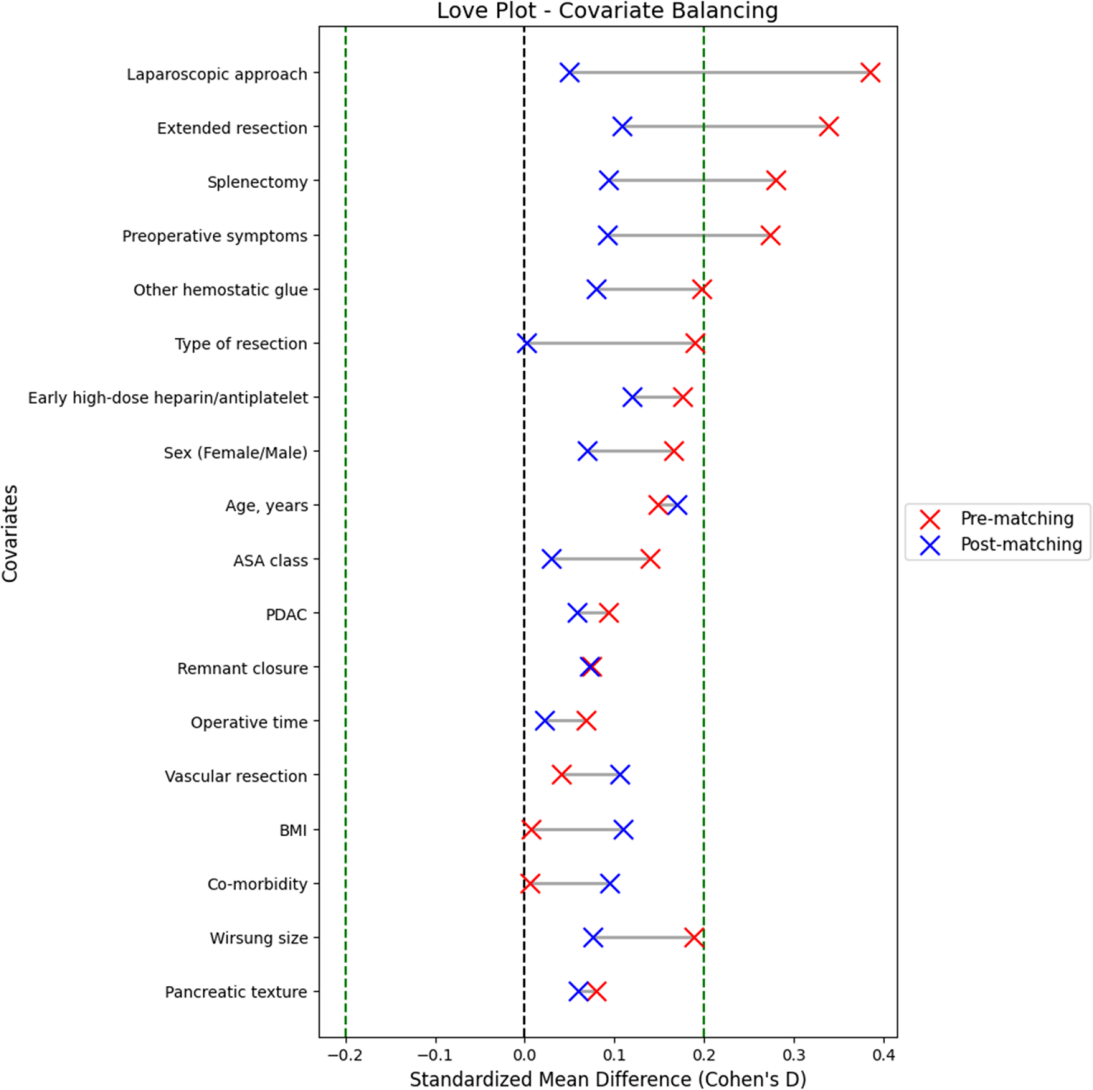
Standardized mean differences (SMD) for baseline covariates before and after inverse probability of treatment weighting (IPTW) adjustment. The dashed line indicates the threshold for clinically relevant imbalance (SMD = 0.2)

Table 1 shows the patients before and after IPTW adjustment. Several covariates exhibit baseline imbalance, with standardized mean differences (SMDs) exceeding the non-small threshold (> 0.2). These included surgical approach (laparoscopic vs. open, SMD = 0.385), splenectomy (yes vs. no, SMD = 0.279), and preoperative symptoms (yes vs. no, SMD = 0.273). The use of other hemostatic agents (SMD = 0.197) and sex distribution (male vs. female, SMD = 0.166) were close to the small-to-moderate range. After IPTW adjustment, all covariates had SMDs < 0.2, with most ≤ 0.1, indicating adequate balance between treatment groups (Fig. 1).

**Table 1:** Patients included in the study

Parameters	No ThacoSil^@^	ThacoSil^@^	SMD	
	N(%) or median (IQR)	N(%) or median (IQR)	pre-matching	post-matching
Age, years	66 (55–75)	70 (54–77)	0.148	0.169
Sex				
Female	95 (56.6)	27 (48.2)	0.166	0.070
Male	73 (43.4)	29 (51.8)		
Co-mordibity				
No	62 (36.9)	19 (33.9)	0.006	0.095
Yes	106 (63.1)	37 (66)		
Preoperative symptoms				
No	95 (56.6)	39 (69.6)	0.273	0.092
Yes	73 (43.4)	17 (30.4)		
BMI, Kg/m^2^	25 (22–28)	25 (23–28)	0.007	0.109
ASA class				
I	3 (1.8)	1 (1.8)	0.139	0.030
II	66 (39.3)	27 (48.2)		
III	98 (58.3)	27 (48.2)		
IV	1 (0.6)	1 (1.8)		
Wirsung size, mm	3 (3–3)	3 (3–6)	0.188	0.076
Need of early use of high dose of heparin or antiplatelet				
No	151 (89.9)	47 (83.9)	0.176	0.119
Yes	17 (10.1)	9 (16)		
Type of resection				
Body-tail resection	163 (97)	52 (92.9)	0.190	0.002
Neck-body-tail resection	5 (3)	4 (7.1)		
Splenectomy				
No	18 (10.7)	2 (3.6)	0.279	0.093
Yes	150 (89.3)	54 (96.4)		
Vascular resection				
No	164 (97.6)	55 (98.2)	0.041	0.106
Yes	4 (2.4)	1 (1.8)		
Extended resection				
No	152 (90.5)	55 (98.2)	0.338	0.108
Yes	16 (9.5)	1 (1.8)		
Thickeness of pancreatic remnant				
Soft	126 (75)	40 (71.4)	0.080	0.060
Hard	42 (25)	16 (28.6)		
Remnant closure				
Hand sewn	63 (37.5)	19 (33.9)	0.074	0.072
Linear stapler	105 (62.5)	37 (66.1)		
Operative time, min	237 (187–282)	220 (184–295)	0.068	0.022
Laparoscopic approach				
No	79 (47)	16 (28.6)	0.385	0.049
Yes	89 (53)	40 (71.4)		
Other hemostatic glue				
No	115 (68.5)	33 (58.9)	0.197	0.079
Yes	53 (31.5)	23 (41.1)		
PDAC				
No	127 (75.6)	40 (71.4)	0.093	0.058
Yes	41 (24.4)	16 (28.6)		

Legend: *IQR* interquartile range, *SMD* Standardized mean difference; the risk of bias was: small, for d-value between 0 to 0.2 (percentage of non-overlap population ≤15%); moderate for d-value >0.2 to 0.5 medium (percentage of non-overlap population ≤33 %); large for d-value >0.50 to 0.80 large, percentage of non-overlap population ≤50%); very large for d-value> 0.8 (percentage of non-overlap population >50 %); *BMI* Body mass index, *ASA* American Anesthesiologist Association score, *PDAC* Pancreatic adenocarcinoma

The PPH rate was 8%, and among these events, 16 (89%) were late hemorrhages, and 2 (11%) were early. According to ISGPS grading, 1 case (6%) was grade A, 8 cases (44%) grade B, and 9 cases (50%) grade C. The POPF rate was 33.9%, the median CCI was 8.7 (0–20.9), the reoperation rate was 3.6%, and the 90-day mortality rate was 0.9%. The median length of stay (LOS) was 10 days (7–13).

### IPTW-weighted analysis

In the weighted analysis (Table 2), the risk difference (RD) for postpancreatectomy hemorrhage (PPH) was − 0.065 (95% CI − 0.121 to − 0.009; *p* = 0.014), with a number needed to treat (NNT) of 15 (95% CI 8–116) and an E-value of 6.7. The RD for clinically relevant POPF was 0.116 (95% CI − 0.044 to 0.277; *p* = 0.105). The mean difference (MD) for the Comprehensive Complication Index (CCI) was 3.459 (95% CI − 1.596 to 8.515; *p* = 0.091). The RD for reoperation was − 0.016 (95% CI − 0.068 to 0.036; *p* = 0.486), for 90-day mortality 0.012 (95% CI − 0.025 to 0.049; *p* = 0.498), and the MD for length of stay (LOS) was − 0.242 days (95% CI − 2.658 to 2.175; *p* = 0.856).

**Table 2:** Results in overall cohort

Outcomes	No ThacoSil^@^	ThacoSil^@^	RD or MD (95CI)	*p*-value	NNT (95 CI)	e-value
PPH (yes)	16/168 (9.5)	2/56 (3.6)	-0.065 (-0.121 to -0.009)	0.014	15 (8 to 116)	6.7
POPF (yes)	51/168 (30.4)	25/56 (44.6)	0.116 (-0.044 to 0.277)	0.105	9* (22 to 4*)	2.1
CCI	8.7 (0 to 20.9)	10.4 (8.7 to 21.7)	3.459 (-1.596 to 8.515)	0.091	-	-
Reoperation (yes)	7/168 (4.2)	1/56 (1.8)	-0.016 (-0.068 to 0.036)	0.486	61 (14 to 28*)	2.9
90-day mortality (yes)	1/167 (0.6)	1/56 (1.8)	0.012 (-0.025 to 0.049)	0.498	82* (39 to 20*)	5.7
LOS, day	10 (7 to 13)	9 (7 to 13)	-0.242 (-2.658 to 2.175)	0.856	-	-

Legenda: *RD* Risk difference, *MD* Mean Difference, *CI* Confidence Interval, *NNT* number needed to treat; *=NNT became NNH for presence of risk increase; POPF =Clinically relevant postoperative pancreatic fistula; *PPH* Postpancreatectomy hemorrhage, *POPF* Clinically relevant post-operative pancreatic fistula, *CCI* Comprehensive Complication Index, *LOS* Length of Stay, *NE* not estimable for absence of event in one arm; - = not computable

Table 3 shows the stratified analysis for POPF. In patients without clinically relevant POPF, the RD for PPH was − 0.013 (95% CI − 0.063 to 0.036; *p* = 0.369; E-value 2.8), the MD for CCI was 5.909 (95% CI − 1.416 to 13.234; *p* = 0.063), the RD for reoperation was − 0.019 (95% CI − 0.045 to 0.007; *p* = 0.121), for 90-day mortality 0.033 (95% CI − 0.031 to 0.098; *p* = 0.510), and the MD for LOS was − 0.205 days (95% CI − 1.862 to 1.453; *p* = 0.759).

**Table 3:** Results stratified by POPF risk

Outcomes	POPF, no				POPF, yes			
	RD or MD (95 CI)	*p*-value	NNT (95 CI)	e-value	RD or MD (95 CI)	*p*-value	NNT (95 CI)	e-value
PPH	-0.013 (-0.063 to 0.036)	0.369	75 (16 to 28*)	2.8	-0.180 (-0.311 to -0.048)	0.004	6 (3 to 21)	12.6
CCI	5.909 (-1.416 to 13.234)	0.063	-	-	-4.587 (-10.364 to 1.190)	0.078	-	-
Reoperation	-0.019 (-0.045 to 0.007)	0.121	53 (22 to 138*)	NE	-0.029 (-0.150 to 0.092)	0.596	35 (7 to 11*)	2.5
90-day mortality	0.033 (-0.031 to 0.098)	0.510	30* (31 to 10*)	NE	-0.018 (-0.055 to 0.018)	0.075	53 (18 to 56*)	NE
LOS	-0.205 (-1.862 to 1.453)	0.759	-	-	-2.083 (-7.039 to 2.872)	0.712	-	-

Legenda: *RD* Risk difference, *MD* Mean Difference, *CI* Confidence Interval, *NNT* number needed to treat;*= NNT becomes NNH when risk increase; POPF =Clinically relevant postoperative pancreatic fistula; PPH= Postpancreatectomy hemorrhage; POPF= Clinically relevant post-operative pancreatic fistula; CCI= Comprehensive Complication Index; *LOS* Length of Stay, *NE* not estimable for absence of event in one arm; - = not computable

**Fig. 2:**
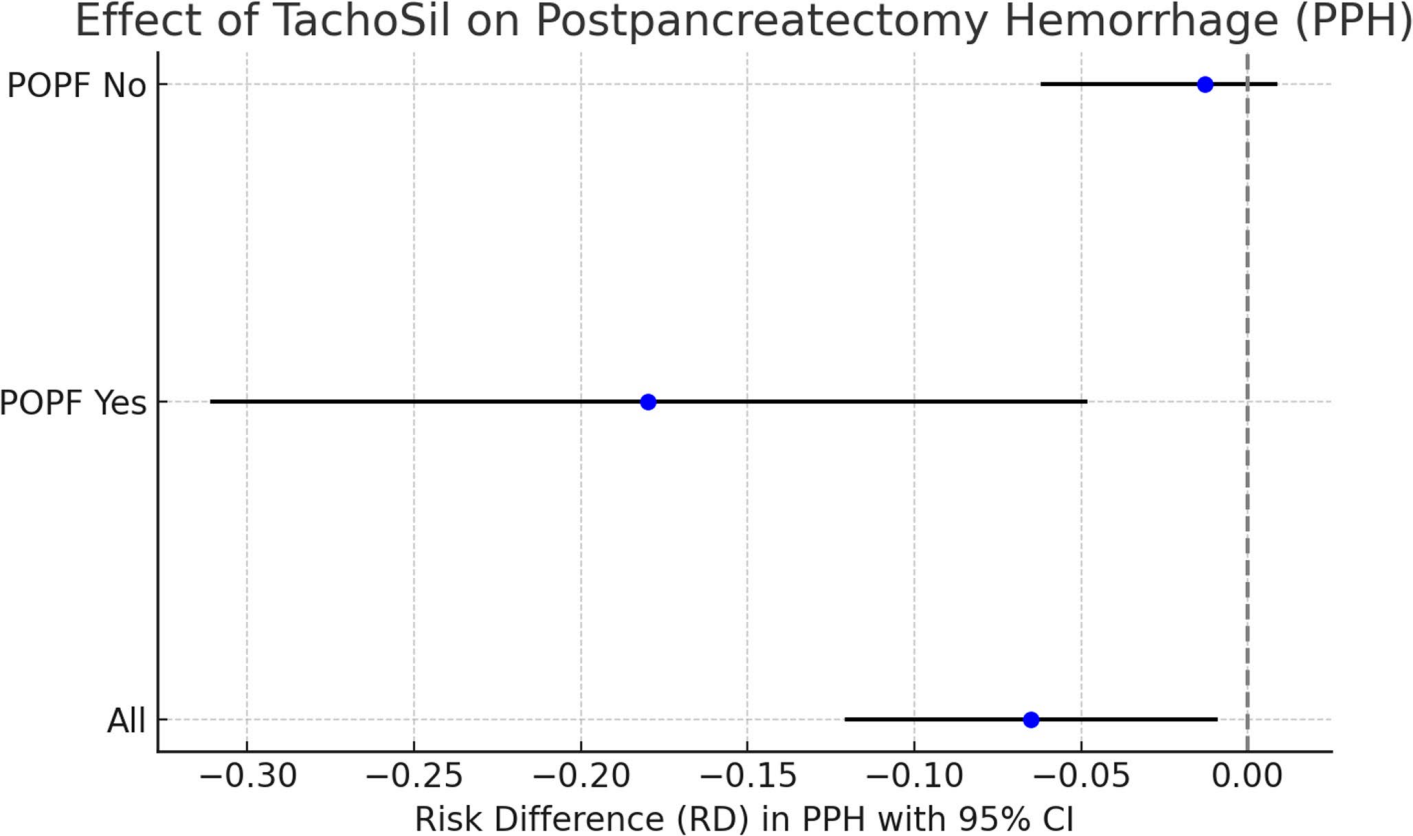
Incidence of postpancreatectomy hemorrhage (PPH) in patients undergoing distal pancreatectomy with or without TachoSil^®^, in the overall cohort and stratified by the presence of clinically relevant postoperative pancreatic fistula (POPF)

In patients with clinically relevant POPF, the RD for PPH was − 0.180 (95% CI − 0.311 to − 0.048; *p* = 0.004; E-value 12.6), the MD for CCI was − 4.587 (95% CI − 10.364 to 1.190; *p* = 0.078), the RD for reoperation was − 0.029 (95% CI − 0.150 to 0.092; *p* = 0.596; E-value 2.5), for 90-day mortality − 0.018 (95% CI − 0.055 to 0.018; *p* = 0.075), and the MD for LOS was − 2.083 days (95% CI − 7.039 to 2.872; *p* = 0.712). Figure 2 shows the distribution of PPH incidence across groups in the overall population and by POPF status.

## Discussion

In this IPTW-adjusted observational study, the use of TachoSil^®^ after DP was associated with a statistically significant reduction in postpancreatectomy hemorrhage (PPH) in the overall population and, more markedly, among patients who developed clinically relevant postoperative pancreatic fistula (POPF). No significant differences were observed for the incidence of POPF itself, the Comprehensive Complication Index (CCI), reoperation rate, 90-day mortality, or postoperative length of stay (LOS). These findings suggested that TachoSil^®^ may not influence the occurrence of POPF, but it could mitigate one of its most severe and clinically impactful sequelae, such as PPH. Our results align partially with those of the pivotal randomized controlled trial by Montorsi et al.^8^, which found no significant reduction in overall POPF rates with TachoSil^®^ use after DP.

That trial, conducted before the International Study Group of Pancreatic Surgery (ISGPS) definition for PPH was established [4], did not systematically assess bleeding events as a primary outcome. As a result, the potential benefit of TachoSil^®^ in preventing PPH, particularly in the presence of POPF, may have been underappreciated. The present study, applying standardized ISGPS criteria for PPH and contemporary analytic methods, provides evidence supporting a targeted protective effect in high-risk patients. The observed NNT of 15 for PPH prevention in the overall cohort, and of only 5 in the POPF subgroup, represents a clinically relevant magnitude of effect, particularly given the high morbidity and mortality associated with post-pancreatectomy bleeding. [7, 8] From a pathophysiological standpoint, the mechanism underlying the observed benefit may relate to the capacity of TachoSil^®^ to provide immediate hemostatic sealing of the pancreatic stump [14], potentially reducing early exposure of adjacent vascular structures to proteolytic pancreatic juice. Indeed, the breakdown of tissue planes and persistent enzymatic exposure increase the risk of vascular erosion and pseudoaneurysm formation, which are common precursors to delayed hemorrhage. [6] The interaction between POPF and PPH is well known [6] and confirmed by e-values. E-value increased moving from the entire cohort (6.7) to the POPF subgroup (12.7). In other words, considering POPF as the main covariate related to PPH, the benefit effect of TachoSil^®^ is more evident and more robust.

The selective use of TachoSil^®^ in patients with a significant risk of POPF could be one part of a multilevel strategy in preventing the detrimental effects of POPF. Indeed, with the D-FRS, it is now possible at the time of surgery to identify patients at significant risk for postoperative pancreatic fistula. [15].

The present study has strengths and limitations. It is based on a prospectively maintained, single-center database from a high-volume pancreatic surgery unit, ensuring detailed and consistent perioperative data collection. The use of IPTW enabled robust adjustment for measured confounders and achieved excellent post-weighting covariate balance, thereby reducing selection bias inherent in observational designs. Endpoints were defined according to standardized ISGPS criteria, enabling comparability with contemporary literature. Nevertheless, limitations must be acknowledged. The observational design precludes definitive causal inference, and residual confounding from unmeasured variables cannot be excluded despite the robustness suggested by E-values. The single-center nature of the study may limit generalizability to settings with different case mixes, surgical techniques, or postoperative care protocols. Additionally, the relatively small number of PPH events, especially within stratified subgroups, may have limited the statistical power and reduced the precision of effect estimates. This is reflected in the wide confidence intervals observed for several outcomes, highlighting that some findings should be interpreted cautiously and considered exploratory rather than definitive. Moreover, while our stratified analysis by POPF occurrence is exploratory and hypothesis-generating, it provides insight into the mechanistic pathway leading to PPH. In clinical practice, surgeons rely on preoperative or intraoperative risk scores such as D-FRS for decision-making, but analyzing outcomes according to observed POPF allows us to explore whether TachoSil^®^ may be particularly protective in the context of fistula-related hemorrhage. Future research should aim to validate these findings in larger, multicenter cohorts and ideally through randomized trials incorporating standardized PPH definitions as the primary endpoint. By randomizing only patients at high risk according to the D-FRS (> 25% predicted POPF incidence), and assuming a control group PPH rate of 20%, a superiority trial powered to detect an absolute risk reduction of 15% (α = 0.05, 90% power) would require 99 patients per arm ( near 198 total), whereas an effect size consistent with our pilot data (absolute reduction of 18%) would require 62 patients per arm (124 total). In conclusion, this study suggests that TachoSil use in distal pancreatectomy is associated with a significant reduction in PPH, particularly in patients with clinically relevant POPF, without affecting POPF incidence itself. These findings support a paradigm in which adjunctive measures are evaluated not only for their effect on leak rates but also for their ability to prevent severe, downstream complications. Incorporating PPH prevention into surgical decision-making and trial design could enhance patient safety and improve outcomes after distal pancreatectomy.

## Data Availability

Dataset are available upon request from the authors by email.
